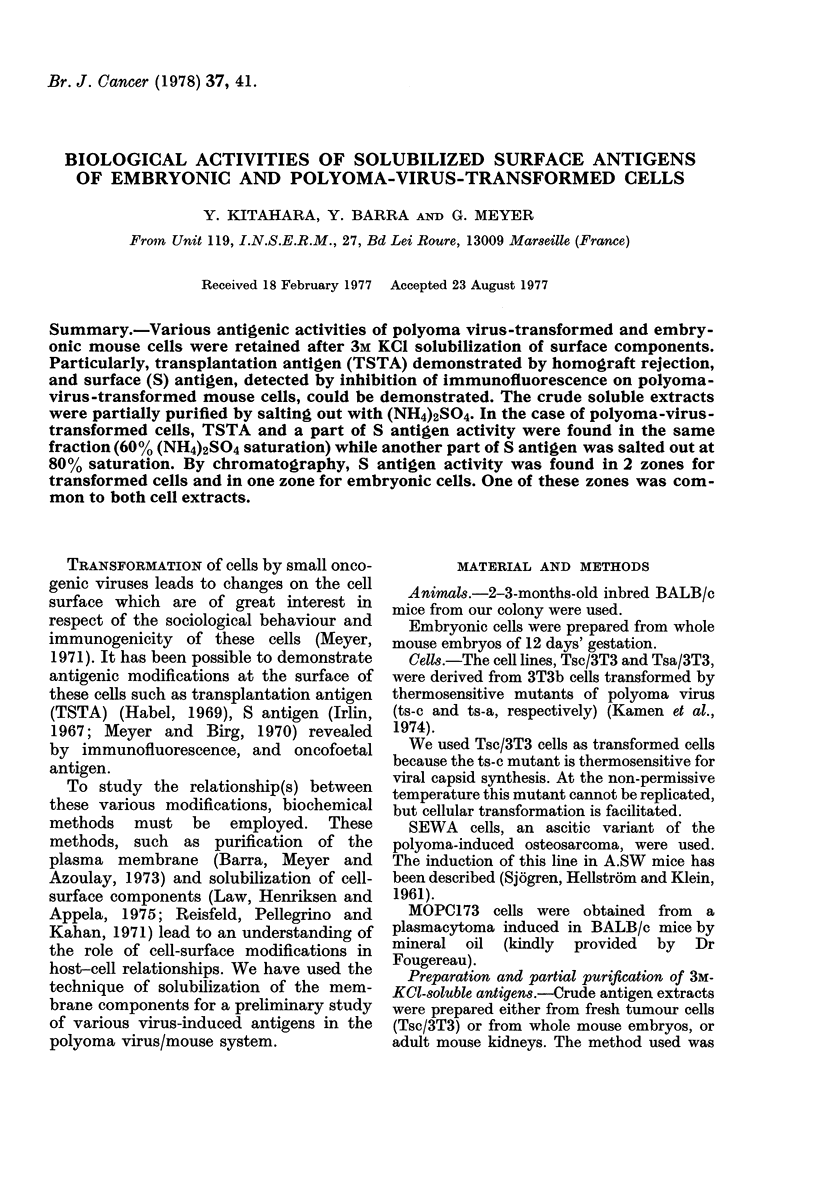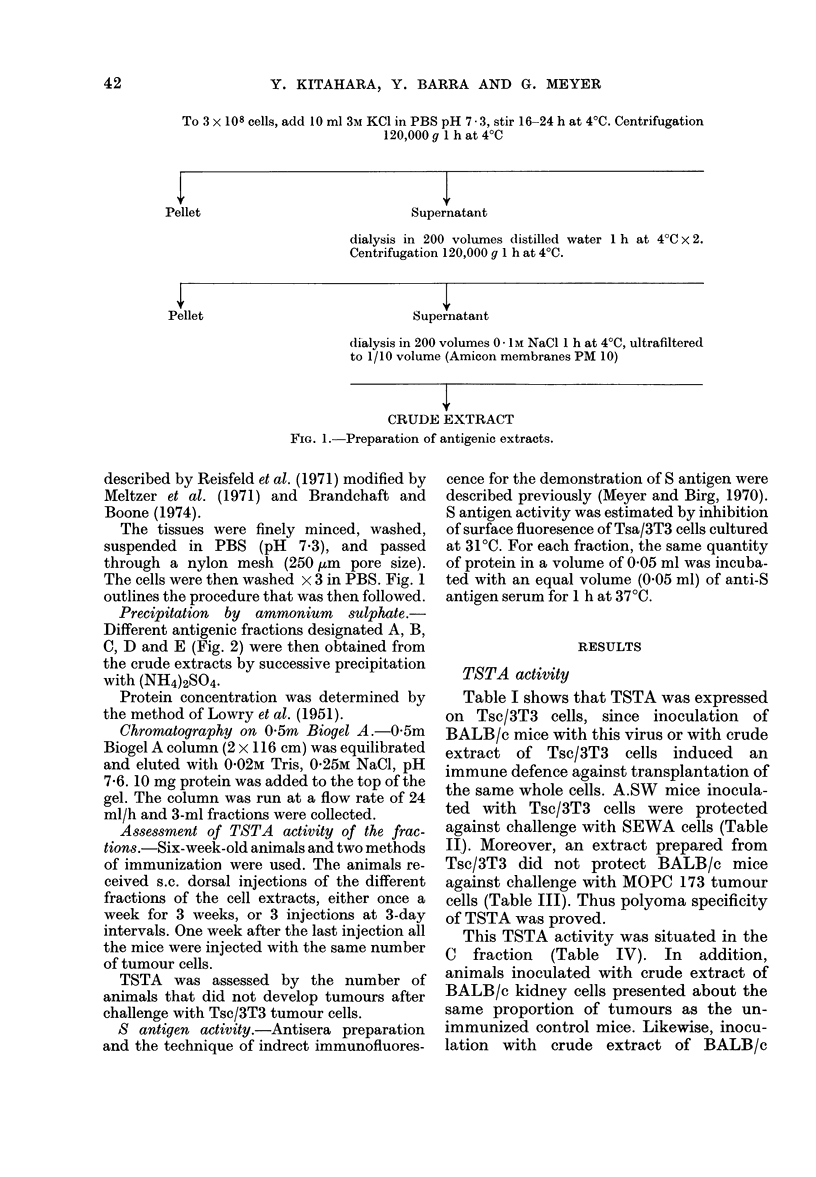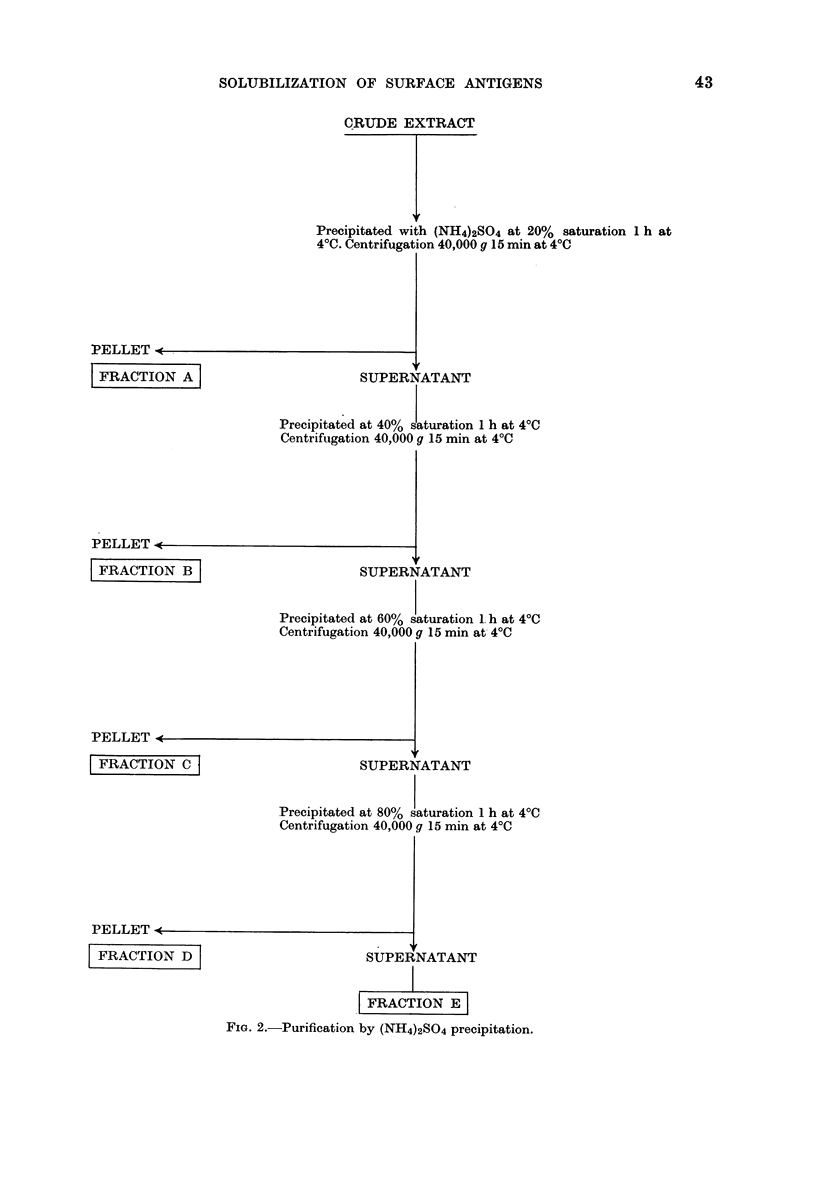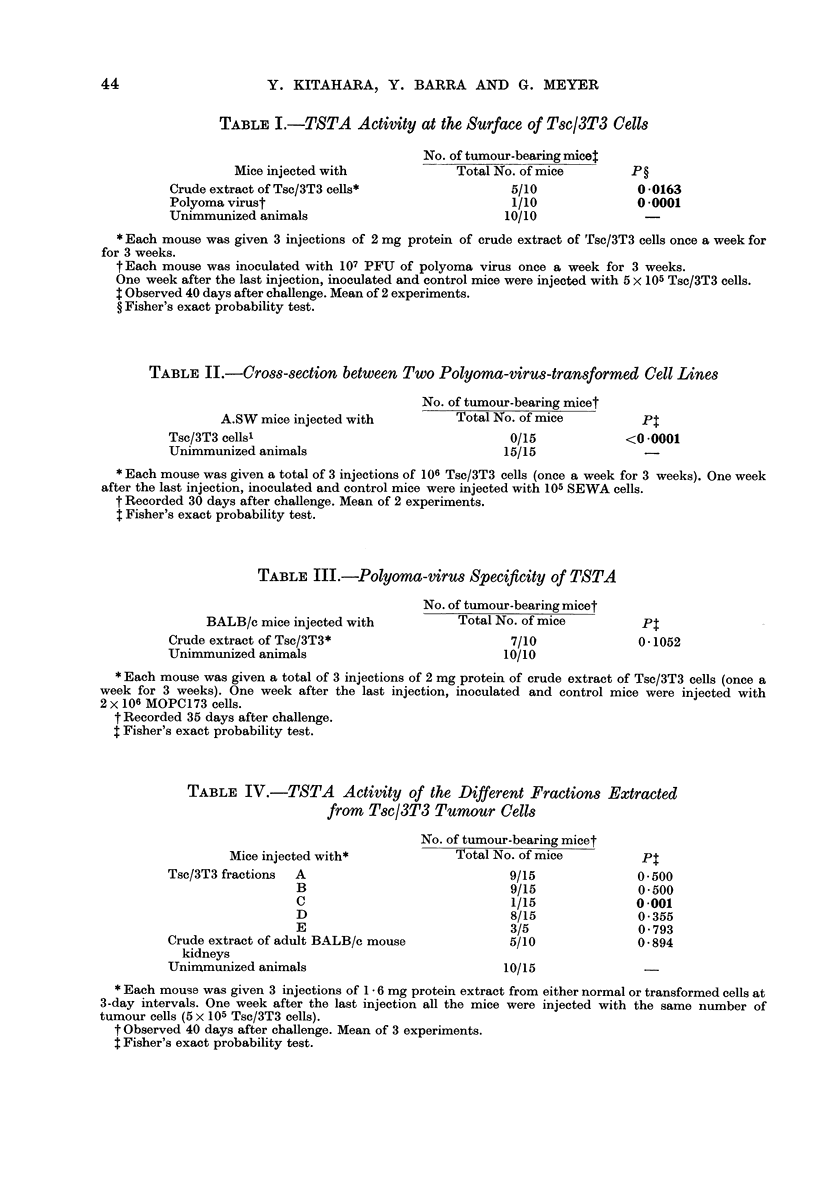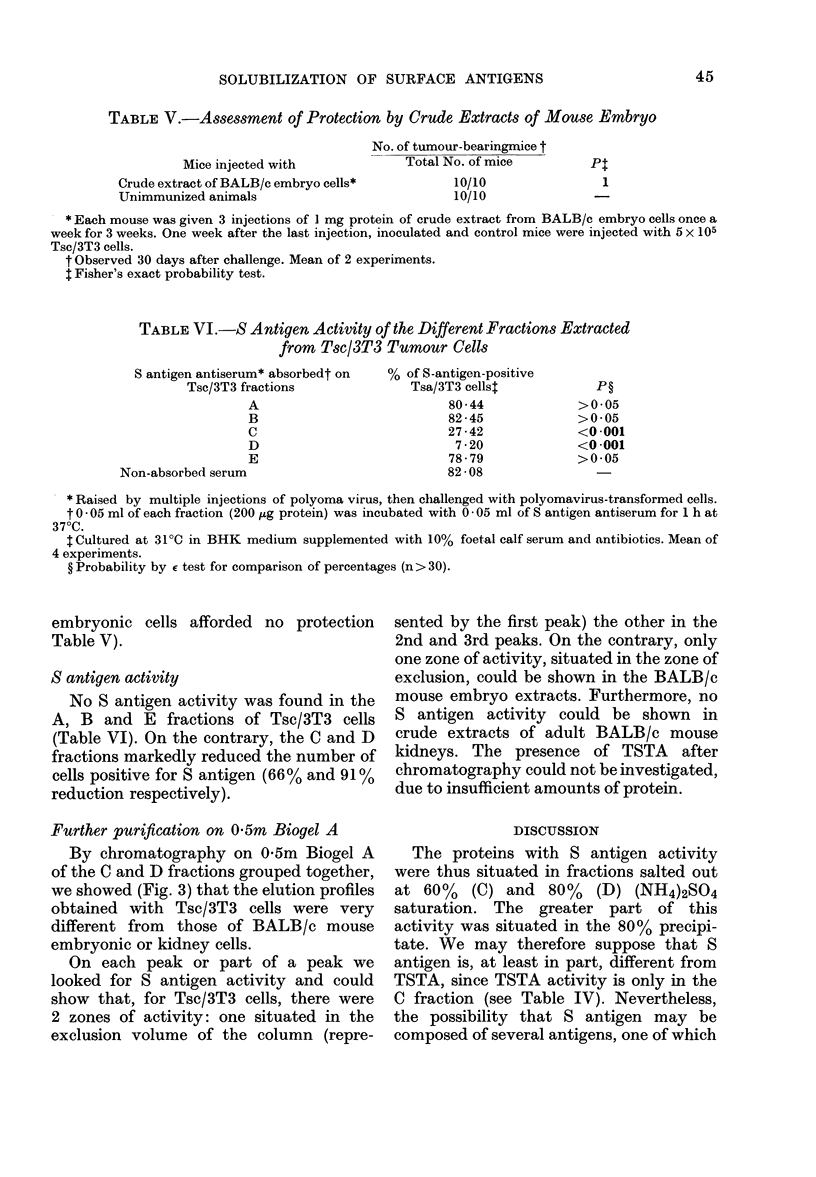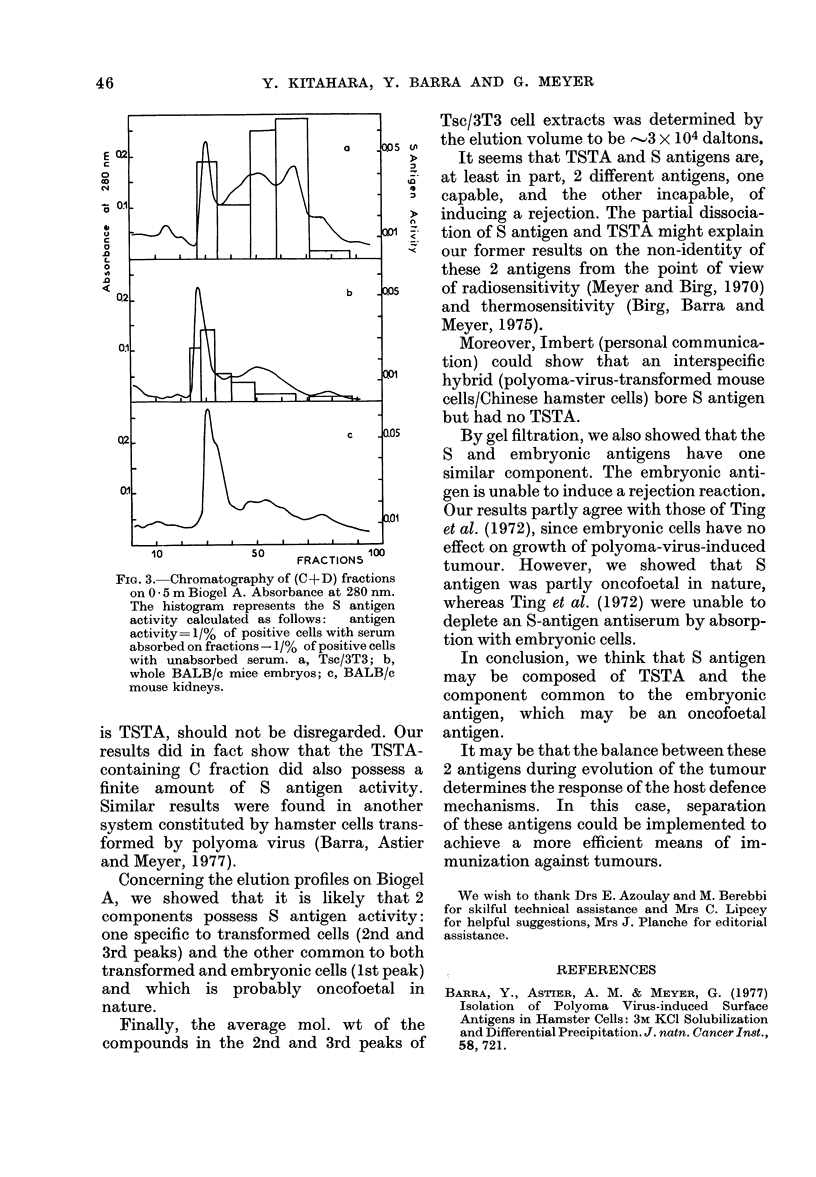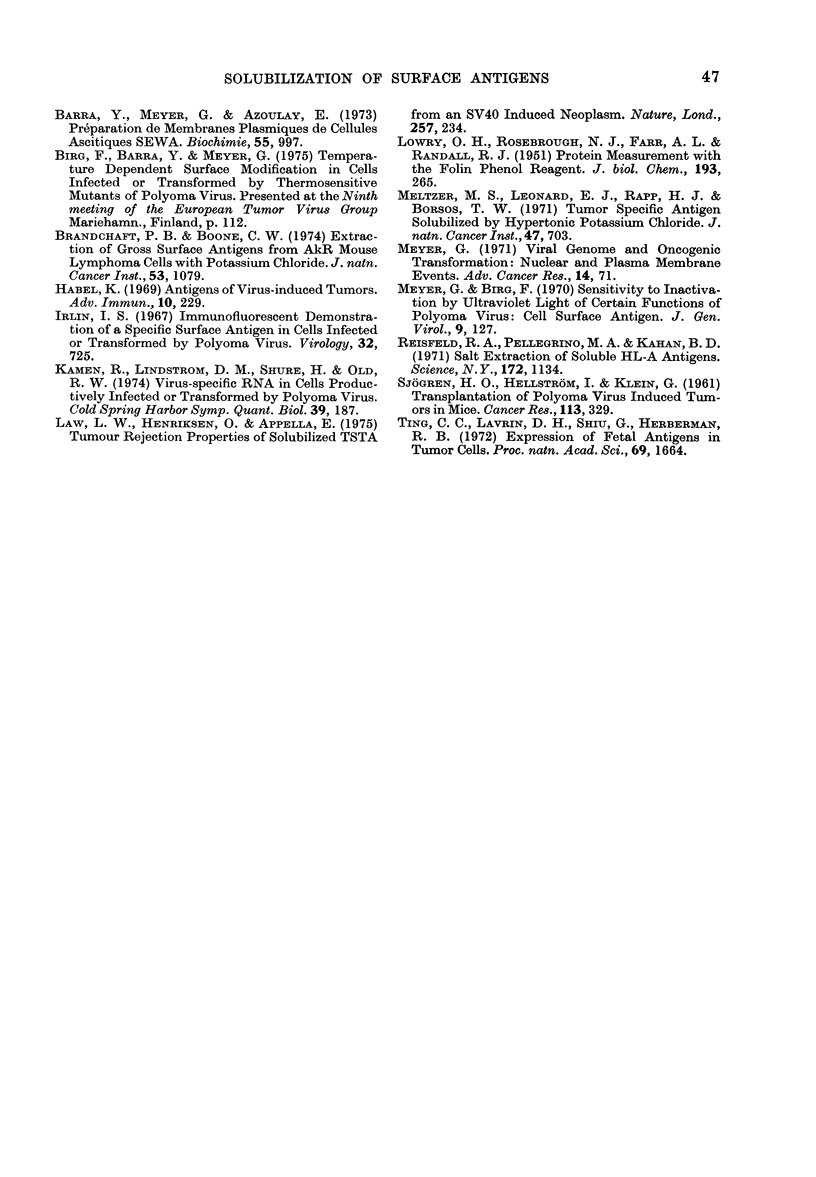# Biological activities of solubilized surface antigens of embryonic and polyoma-virus-transformed cells.

**DOI:** 10.1038/bjc.1978.6

**Published:** 1978-01

**Authors:** Y. Kitahara, Y. Barra, G. Meyer

## Abstract

Various antigenic activities of polyoma virus-transformed and embryonic mouse cells were retained after 3M KCl solubilization of surface components. Particularly, transplantation antigen (TSTA) demonstrated by homograft rejection, and surface (S) antigen, detected by inhibition of immunofluorescence on polyoma-virus-transformed mouse cells, could be demonstrated. The crude soluble extracts were partially purified by salting out with (NH4)2SO4. In the case of polyoma-virus-transformed cells, TSTA and a part of S antigen activity were found in the same fraction (60% (NH4)2SO4 saturation) while another part of S antigen was salted out at 80% saturation. By chromatography, S antigen activity was found in 2 zones for transformed cells and in one zone for embryonic cells. One of these zones was common to both cell extracts.


*Br. J. Cancer* (1978) **37**, 41.

### BIOLOGICAL ACTIVITIES OF SOLUBILIZED SURFACE ANTIGENS OF EMBRYONIC AND POLYOMA-VIRUS-TRANSFORMED CELLS

Y. KITAHARA, Y. BARRA AND G. MEYER

*From Unit* 119, *I.N.S.E.R.M.*, 27, *Bd Lei Roure*, 13009 *Marseille* (*France*)

Received 18 February 1977 Accepted 23 August 1977

**Summary.—Various antigenic activities of polyoma virus-transformed and embryonic mouse cells were retained after 3M KCl solubilization of surface components. Particularly, transplantation antigen (TSTA) demonstrated by homograft rejection, and surface (S) antigen, detected by inhibition of immunofluorescence on polyoma-virus-transformed mouse cells, could be demonstrated. The crude soluble extracts were partially purified by salting out with $(NH_4)_2SO_4$. In the case of polyoma-virus-transformed cells, TSTA and a part of S antigen activity were found in the same fraction (60% $(NH_4)_2SO_4$ saturation) while another part of S antigen was salted out at 80% saturation. By chromatography, S antigen activity was found in 2 zones for transformed cells and in one zone for embryonic cells. One of these zones was common to both cell extracts.**

TRANSFORMATION of cells by small oncogenic viruses leads to changes on the cell surface which are of great interest in respect of the sociological behaviour and immunogenicity of these cells (Meyer, 1971). It has been possible to demonstrate antigenic modifications at the surface of these cells such as transplantation antigen (TSTA) (Habel, 1969), S antigen (Irlin, 1967; Meyer and Birg, 1970) revealed by immunofluorescence, and oncofoetal antigen.

To study the relationship(s) between these various modifications, biochemical methods must be employed. These methods, such as purification of the plasma membrane (Barra, Meyer and Azoulay, 1973) and solubilization of cell-surface components (Law, Henriksen and Appela, 1975; Reisfeld, Pellegrino and Kahan, 1971) lead to an understanding of the role of cell-surface modifications in host–cell relationships. We have used the technique of solubilization of the membrane components for a preliminary study of various virus-induced antigens in the polyoma virus/mouse system.

### MATERIAL AND METHODS

*Animals*.—2–3-months-old inbred BALB/c mice from our colony were used.

Embryonic cells were prepared from whole mouse embryos of 12 days' gestation.

*Cells*.—The cell lines, Tsc/3T3 and Tsa/3T3, were derived from 3T3b cells transformed by thermosensitive mutants of polyoma virus (ts-c and ts-a, respectively) (Kamen *et al.*, 1974).

We used Tsc/3T3 cells as transformed cells because the ts-c mutant is thermosensitive for viral capsid synthesis. At the non-permissive temperature this mutant cannot be replicated, but cellular transformation is facilitated.

SEWA cells, an ascitic variant of the polyoma-induced osteosarcoma, were used. The induction of this line in A.SW mice has been described (Sjögren, Hellström and Klein, 1961).

MOPC173 cells were obtained from a plasmacytoma induced in BALB/c mice by mineral oil (kindly provided by Dr Fougereau).

*Preparation and partial purification of* 3M-*KCl-soluble antigens*.—Crude antigen extracts were prepared either from fresh tumour cells (Tsc/3T3) or from whole mouse embryos, or adult mouse kidneys. The method used was

FIG. 1.—Preparation of antigenic extracts.

described by Reisfeld *et al.* (1971) modified by Meltzer *et al.* (1971) and Brandchaft and Boone (1974).

The tissues were finely minced, washed, suspended in PBS (pH 7·3), and passed through a nylon mesh (250 $\mu$m pore size). The cells were then washed $\times 3$ in PBS. Fig. 1 outlines the procedure that was then followed.

*Precipitation by ammonium sulphate.*—Different antigenic fractions designated A, B, C, D and E (Fig. 2) were then obtained from the crude extracts by successive precipitation with $(NH_4)_2SO_4$.

Protein concentration was determined by the method of Lowry *et al.* (1951).

*Chromatography on 0·5m Biogel A.*—0·5m Biogel A column ($2 \times 116$ cm) was equilibrated and eluted with 0·02M Tris, 0·25M NaCl, pH 7·6. 10 mg protein was added to the top of the gel. The column was run at a flow rate of 24 ml/h and 3-ml fractions were collected.

*Assessment of TSTA activity of the fractions.*—Six-week-old animals and two methods of immunization were used. The animals received s.c. dorsal injections of the different fractions of the cell extracts, either once a week for 3 weeks, or 3 injections at 3-day intervals. One week after the last injection all the mice were injected with the same number of tumour cells.

TSTA was assessed by the number of animals that did not develop tumours after challenge with Tsc/3T3 tumour cells.

*S antigen activity.*—Antisera preparation and the technique of indrect immunofluorescence for the demonstration of S antigen were described previously (Meyer and Birg, 1970). S antigen activity was estimated by inhibition of surface fluoresence of Tsa/3T3 cells cultured at 31°C. For each fraction, the same quantity of protein in a volume of 0·05 ml was incubated with an equal volume (0·05 ml) of anti-S antigen serum for 1 h at 37°C.

### RESULTS

### *TSTA activity*

Table I shows that TSTA was expressed on Tsc/3T3 cells, since inoculation of BALB/c mice with this virus or with crude extract of Tsc/3T3 cells induced an immune defence against transplantation of the same whole cells. A.SW mice inoculated with Tsc/3T3 cells were protected against challenge with SEWA cells (Table II). Moreover, an extract prepared from Tsc/3T3 did not protect BALB/c mice against challenge with MOPC 173 tumour cells (Table III). Thus polyoma specificity of TSTA was proved.

This TSTA activity was situated in the C fraction (Table IV). In addition, animals inoculated with crude extract of BALB/c kidney cells presented about the same proportion of tumours as the unimmunized control mice. Likewise, inoculation with crude extract of BALB/c

FIG. 2.—Purification by $(NH_4)_2SO_4$ precipitation.

TABLE I.—*TSTA Activity at the Surface of Tsc/3T3 Cells*

| Mice injected with | No. of tumour-bearing mice‡ / Total No. of mice | P§ |
|---|---|---|
| Crude extract of Tsc/3T3 cells* | 5/10 | **0·0163** |
| Polyoma virus† | 1/10 | **0·0001** |
| Unimmunized animals | 10/10 | — |

* Each mouse was given 3 injections of 2 mg protein of crude extract of Tsc/3T3 cells once a week for for 3 weeks.

† Each mouse was inoculated with $10^7$ PFU of polyoma virus once a week for 3 weeks.

One week after the last injection, inoculated and control mice were injected with $5 \times 10^5$ Tsc/3T3 cells.

‡ Observed 40 days after challenge. Mean of 2 experiments.

§ Fisher's exact probability test.

TABLE II.—*Cross-section between Two Polyoma-virus-transformed Cell Lines*

| A.SW mice injected with | No. of tumour-bearing mice† / Total No. of mice | P‡ |
|---|---|---|
| Tsc/3T3 cells[1] | 0/15 | **<0·0001** |
| Unimmunized animals | 15/15 | — |

* Each mouse was given a total of 3 injections of $10^6$ Tsc/3T3 cells (once a week for 3 weeks). One week after the last injection, inoculated and control mice were injected with $10^5$ SEWA cells.

† Recorded 30 days after challenge. Mean of 2 experiments.

‡ Fisher's exact probability test.

TABLE III.—*Polyoma-virus Specificity of TSTA*

| BALB/c mice injected with | No. of tumour-bearing mice† / Total No. of mice | P‡ |
|---|---|---|
| Crude extract of Tsc/3T3* | 7/10 | 0·1052 |
| Unimmunized animals | 10/10 | |

* Each mouse was given a total of 3 injections of 2 mg protein of crude extract of Tsc/3T3 cells (once a week for 3 weeks). One week after the last injection, inoculated and control mice were injected with $2 \times 10^6$ MOPC173 cells.

† Recorded 35 days after challenge.

‡ Fisher's exact probability test.

TABLE IV.—*TSTA Activity of the Different Fractions Extracted from Tsc/3T3 Tumour Cells*

| Mice injected with* | | No. of tumour-bearing mice† / Total No. of mice | P‡ |
|---|---|---|---|
| Tsc/3T3 fractions | A | 9/15 | 0·500 |
| | B | 9/15 | 0·500 |
| | C | 1/15 | **0·001** |
| | D | 8/15 | 0·355 |
| | E | 3/5 | 0·793 |
| Crude extract of adult BALB/c mouse kidneys | | 5/10 | 0·894 |
| Unimmunized animals | | 10/15 | — |

* Each mouse was given 3 injections of 1·6 mg protein extract from either normal or transformed cells at 3-day intervals. One week after the last injection all the mice were injected with the same number of tumour cells ($5 \times 10^5$ Tsc/3T3 cells).

† Observed 40 days after challenge. Mean of 3 experiments.

‡ Fisher's exact probability test.

TABLE V.—*Assessment of Protection by Crude Extracts of Mouse Embryo*

| Mice injected with | No. of tumour-bearingmice † / Total No. of mice | P‡ |
|---|---|---|
| Crude extract of BALB/c embryo cells* | 10/10 | 1 |
| Unimmunized animals | 10/10 | — |

* Each mouse was given 3 injections of 1 mg protein of crude extract from BALB/c embryo cells once a week for 3 weeks. One week after the last injection, inoculated and control mice were injected with $5 \times 10^5$ Tsc/3T3 cells.
† Observed 30 days after challenge. Mean of 2 experiments.
‡ Fisher's exact probability test.

TABLE VI.—*S Antigen Activity of the Different Fractions Extracted from Tsc/3T3 Tumour Cells*

| S antigen antiserum* absorbed† on Tsc/3T3 fractions | % of S-antigen-positive Tsa/3T3 cells‡ | P§ |
|---|---|---|
| A | 80·44 | >0·05 |
| B | 82·45 | >0·05 |
| C | 27·42 | **<0·001** |
| D | 7·20 | **<0·001** |
| E | 78·79 | >0·05 |
| Non-absorbed serum | 82·08 | — |

* Raised by multiple injections of polyoma virus, then challenged with polyomavirus-transformed cells.
† 0·05 ml of each fraction (200 μg protein) was incubated with 0·05 ml of S antigen antiserum for 1 h at 37°C.
‡ Cultured at 31°C in BHK medium supplemented with 10% foetal calf serum and antibiotics. Mean of 4 experiments.
§ Probability by ε test for comparison of percentages (n>30).

embryonic cells afforded no protection Table V).

*S antigen activity*

No S antigen activity was found in the A, B and E fractions of Tsc/3T3 cells (Table VI). On the contrary, the C and D fractions markedly reduced the number of cells positive for S antigen (66% and 91% reduction respectively).

*Further purification on 0·5m Biogel A*

By chromatography on 0·5m Biogel A of the C and D fractions grouped together, we showed (Fig. 3) that the elution profiles obtained with Tsc/3T3 cells were very different from those of BALB/c mouse embryonic or kidney cells.

On each peak or part of a peak we looked for S antigen activity and could show that, for Tsc/3T3 cells, there were 2 zones of activity: one situated in the exclusion volume of the column (represented by the first peak) the other in the 2nd and 3rd peaks. On the contrary, only one zone of activity, situated in the zone of exclusion, could be shown in the BALB/c mouse embryo extracts. Furthermore, no S antigen activity could be shown in crude extracts of adult BALB/c mouse kidneys. The presence of TSTA after chromatography could not be investigated, due to insufficient amounts of protein.

DISCUSSION

The proteins with S antigen activity were thus situated in fractions salted out at 60% (C) and 80% (D) $(NH_4)_2SO_4$ saturation. The greater part of this activity was situated in the 80% precipitate. We may therefore suppose that S antigen is, at least in part, different from TSTA, since TSTA activity is only in the C fraction (see Table IV). Nevertheless, the possibility that S antigen may be composed of several antigens, one of which

FIG. 3.—Chromatography of (C+D) fractions on 0·5 m Biogel A. Absorbance at 280 nm. The histogram represents the S antigen activity calculated as follows: antigen activity = 1/% of positive cells with serum absorbed on fractions — 1/% of positive cells with unabsorbed serum. a, Tsc/3T3; b, whole BALB/c mice embryos; c, BALB/c mouse kidneys.

is TSTA, should not be disregarded. Our results did in fact show that the TSTA-containing C fraction did also possess a finite amount of S antigen activity. Similar results were found in another system constituted by hamster cells transformed by polyoma virus (Barra, Astier and Meyer, 1977).

Concerning the elution profiles on Biogel A, we showed that it is likely that 2 components possess S antigen activity: one specific to transformed cells (2nd and 3rd peaks) and the other common to both transformed and embryonic cells (1st peak) and which is probably oncofoetal in nature.

Finally, the average mol. wt of the compounds in the 2nd and 3rd peaks of Tsc/3T3 cell extracts was determined by the elution volume to be $\sim 3 \times 10^4$ daltons.

It seems that TSTA and S antigens are, at least in part, 2 different antigens, one capable, and the other incapable, of inducing a rejection. The partial dissociation of S antigen and TSTA might explain our former results on the non-identity of these 2 antigens from the point of view of radiosensitivity (Meyer and Birg, 1970) and thermosensitivity (Birg, Barra and Meyer, 1975).

Moreover, Imbert (personal communication) could show that an interspecific hybrid (polyoma-virus-transformed mouse cells/Chinese hamster cells) bore S antigen but had no TSTA.

By gel filtration, we also showed that the S and embryonic antigens have one similar component. The embryonic antigen is unable to induce a rejection reaction. Our results partly agree with those of Ting *et al.* (1972), since embryonic cells have no effect on growth of polyoma-virus-induced tumour. However, we showed that S antigen was partly oncofoetal in nature, whereas Ting *et al.* (1972) were unable to deplete an S-antigen antiserum by absorption with embryonic cells.

In conclusion, we think that S antigen may be composed of TSTA and the component common to the embryonic antigen, which may be an oncofoetal antigen.

It may be that the balance between these 2 antigens during evolution of the tumour determines the response of the host defence mechanisms. In this case, separation of these antigens could be implemented to achieve a more efficient means of immunization against tumours.

We wish to thank Drs E. Azoulay and M. Berebbi for skilful technical assistance and Mrs C. Lipcey for helpful suggestions, Mrs J. Planche for editorial assistance.

### REFERENCES

BARRA, Y., ASTIER, A. M. & MEYER, G. (1977) Isolation of Polyoma Virus-induced Surface Antigens in Hamster Cells: 3M KCl Solubilization and Differential Precipitation. *J. natn. Cancer Inst.*, **58**, 721.

Barra, Y., Meyer, G. & Azoulay, E. (1973) Préparation de Membranes Plasmiques de Cellules Ascitiques SEWA. *Biochimie*, **55**, 997.

Birg, F., Barra, Y. & Meyer, G. (1975) Temperature Dependent Surface Modification in Cells Infected or Transformed by Thermosensitive Mutants of Polyoma Virus. Presented at the *Ninth meeting of the European Tumor Virus Group* Mariehamn., Finland, p. 112.

Brandchaft, P. B. & Boone, C. W. (1974) Extraction of Gross Surface Antigens from AkR Mouse Lymphoma Cells with Potassium Chloride. *J. natn. Cancer Inst.*, **53**, 1079.

Habel, K. (1969) Antigens of Virus-induced Tumors. *Adv. Immun.*, **10**, 229.

Irlin, I. S. (1967) Immunofluorescent Demonstration of a Specific Surface Antigen in Cells Infected or Transformed by Polyoma Virus. *Virology*, **32**, 725.

Kamen, R., Lindstrom, D. M., Shure, H. & Old, R. W. (1974) Virus-specific RNA in Cells Productively Infected or Transformed by Polyoma Virus. *Cold Spring Harbor Symp. Quant. Biol.* **39**, 187.

Law, L. W., Henriksen, O. & Appella, E. (1975) Tumour Rejection Properties of Solubilized TSTA from an SV40 Induced Neoplasm. *Nature, Lond.*, **257**, 234.

Lowry, O. H., Rosebrough, N. J., Farr, A. L. & Randall, R. J. (1951) Protein Measurement with the Folin Phenol Reagent. *J. biol. Chem.*, **193**, 265.

Meltzer, M. S., Leonard, E. J., Rapp, H. J. & Borsos, T. W. (1971) Tumor Specific Antigen Solubilized by Hypertonic Potassium Chloride. *J. natn. Cancer Inst.*, **47**, 703.

Meyer, G. (1971) Viral Genome and Oncogenic Transformation: Nuclear and Plasma Membrane Events. *Adv. Cancer Res.*, **14**, 71.

Meyer, G. & Birg, F. (1970) Sensitivity to Inactivation by Ultraviolet Light of Certain Functions of Polyoma Virus: Cell Surface Antigen. *J. Gen. Virol.*, **9**, 127.

Reisfeld, R. A., Pellegrino, M. A. & Kahan, B. D. (1971) Salt Extraction of Soluble HL-A Antigens. *Science, N.Y.*, **172**, 1134.

Sjögren, H. O., Hellström, I. & Klein, G. (1961) Transplantation of Polyoma Virus Induced Tumors in Mice. *Cancer Res.*, **113**, 329.

Ting, C. C., Lavrin, D. H., Shiu, G., Herberman, R. B. (1972) Expression of Fetal Antigens in Tumor Cells. *Proc. natn. Acad. Sci.*, **69**, 1664.